# Theoretical and experimental approaches to understand the biosynthesis of starch granules in a physiological context

**DOI:** 10.1007/s11120-019-00704-y

**Published:** 2020-01-18

**Authors:** Barbara Pfister, Samuel C. Zeeman, Michael D. Rugen, Robert A. Field, Oliver Ebenhöh, Adélaïde Raguin

**Affiliations:** 1 Department of Biology, Institute of Molecular Plant Biology, ETH Zurich, 8092 Zurich, Switzerland; 2 Department of Biological Chemistry, John Innes Centre, Norwich Research Park, Norwich, NR4 7UH UK; 3 Department of Biology, Institute of Quantitative and Theoretical Biology, Heinrich-Heine University, 40225 Düsseldorf, Germany; 4 Department of Biology, Cluster of Excellence on Plant Sciences, Institute of Quantitative and Theoretical Biology, Heinrich-Heine University, 40225 Düsseldorf, Germany

**Keywords:** Starch, Amylopectin, Biosynthesis, Mathematical modeling

## Abstract

Starch, a plant-derived insoluble carbohydrate composed of glucose polymers, is the principal carbohydrate in our diet and a valuable raw material for industry. The properties of starch depend on the arrangement of glucose units within the constituent polymers. However, key aspects of starch structure and the underlying biosynthetic processes are not well understood, limiting progress towards targeted improvement of our starch crops. In particular, the major component of starch, amylopectin, has a complex three-dimensional, branched architecture. This architecture stems from the combined actions of a multitude of enzymes, each having broad specificities that are difficult to capture experimentally. In this review, we reflect on experimental approaches and limitations to decipher the enzymes’ specificities and explore possibilities for in silico simulations of these activities. We believe that the synergy between experimentation and simulation is needed for the correct interpretation of experimental data and holds the potential to greatly advance our understanding of the overall starch biosynthetic process. We furthermore propose that the formation of glucan secondary structures, concomitant with its synthesis, is a previously overlooked factor that directly affects amylopectin architecture through its impact on enzyme function.

**Electronic supplementary material:**

The online version of this article (10.1007/s11120-019-00704-y) contains supplementary material, which is available to authorized users.

## Introduction

**Fig. 1:**
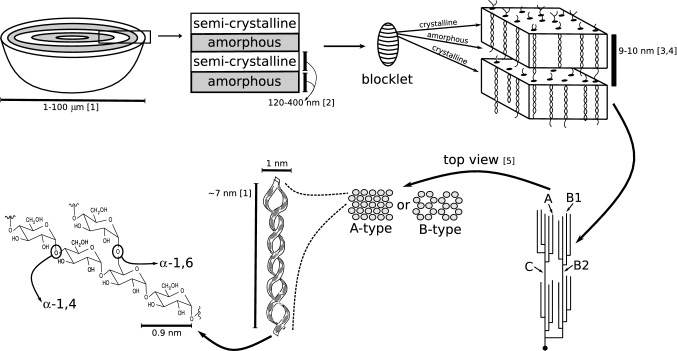
Structure of amylopectin and higher-order arrangements of starch. Two α-1,4/α-1,6-linked glucose polymers, amylopectin and amylose (not shown), comprise starch. Amylopectin is branched with 4–5% α-1,6 linkages, yielding a tree-like structure. This structure has A-chains that are external and unbranched, B-chains that carry other branches, and one C-chain which has the molecule’s single reducing end (indicated with a sphere). Within clusters of linear amylopectin chain segments, double helices form and pack into ordered lamellar arrays, forming either the A- or B-type allomorph, that stack with a 9–10 nm periodicity. This is the basis for the semi-crystalline nature of starch, and amylopectin lamellae are organized into higher-order structures, such as ‘blocklets’ and concentric ‘growth rings’. ([1] Pérez and Bertoft 2010; [2] Yamaguchi et al. 1979; [3] Manners 1989; [4] Jenkins et al. 1993; [5] Imberty et al. 1988)

Starch, the primary energy storage of most plants, is the second most abundant glucose polymer on earth after cellulose and the main source of energy in human diet. Although starch exclusively consists of glucose units that are either α-1,4- or α-1,6-linked, its structure is surprisingly complex. It is composed of two distinct polymers—amylopectin and amylose, which together form massive semi-crystalline granules within the micrometer range (Fig. 1). The arrangement of glucose within starch results in remarkable physico-chemical properties, in particular the capacity to form gels and viscous solutions after solubilization and cooling (Santelia and Zeeman 2011). These properties render starch an in-demand raw material for the food industry, where it functions as a texturizer and thickener, but it also has various non-food applications, for example as an adhesive or in the production of biodegradable plastics and biofuels. The physico-chemical properties depend on numerous starch traits, such as granule size, the ratio between amylopectin and amylose and their fine structure, and the phosphorylation of glucose units within starch (Blennow et al. 2002; Santelia and Zeeman 2011). However, as the functionalities of natural starches often do not meet the industrial requirements, many starches need chemical and/or physical modifications post harvesting, entailing additional costs (Tharanathan 2005). Furthermore, in many cases, the enzymatic processes underlying starch traits are not well understood. Consequently, the genetic improvement of crop plants with the aim of producing starches with desired properties often proceeds via a lengthy trial-and-error process, with a low overall success rate.

Plants produce starch in different tissues, but always in the plastidial compartment—the chloroplasts of leaves and amyloplasts of non-green tissues. In the chloroplast, a proportion of the carbon fixed during photosynthesis by the Calvin–Benson cycle is directly allocated to the formation of starch, while the remainder is exported to the cytosol and used primarily for sucrose production (Stitt and Zeeman 2012). Sucrose is exported from the leaves and its metabolism in sink tissues can serve the formation of starch in the amyloplast, a specialized plastid for the biosynthesis and storage of starch (Smith 2001). It is this storage starch which serves as the primary starch source for human nutrition and industrial applications; it typically accounts for ca. 70% of the dry weight of the harvested parts of our staple crops, including cereal grains, potato tubers, and cassava roots.

In both leaves and non-photosynthetic tissues, starch biosynthesis involves a battery of enzymes that can be categorized into elongating, branching, and debranching. Alongside the starch-producing enzymes, however, most plastids contain other glucan-modifying enzymes like amylases, disproportionating enzyme, and starch phosphorylase (Beck and Ziegler 1989; Zeeman et al. 2010). These enzymes constitute an environment of high degrading capacity, the potential influence of which is frequently overlooked when starch biosynthesis is considered. The interplay between all of these enzymes results in surprising complexity in starch biosynthesis and highlights the importance of the insoluble, granular nature of the starch to ensure its stability.

Leaf starch metabolism is often viewed as a diurnal cycle of synthesis and degradation, in which a substantial amount of starch is synthesized during the day and almost all of it is degraded the subsequent night to support night time metabolism. Despite holding true for a range of species such as Arabidopsis (Gibon et al. 2004), pea (Stitt et al. 1978), or almond trees (Tixier et al. 2018), the extent of starch accumulation and degradation is lower in others and may also depend on the developmental stage of the plant and leaf, growth conditions, and cell type (Trethewey and Smith 2000). Some grasses partition only a low percentage of photoassimilates into leaf starch and store carbohydrates in the form of sucrose or fructans in the vacuoles of mesophyll cells (Trethewey and Smith 2000). In tobacco, the magnitude of diurnal changes in leaf starch content lessens with leaf maturation, and fully expanded leaves retain relatively high levels of starch during day and night (Matheson and Wheatley 1963), suggesting a lower demand of starch reserves at this developmental stage.

In Arabidopsis, the rates of starch synthesis and degradation appear to be tightly linked to light conditions: if days are shorter, the daytime synthesis rate increases, while the night time degradation rate decreases, and vice versa (Graf et al. 2010; Pokhilko et al. 2014). These dynamic adjustments prevent the plant from exhausting its energy reserves before dawn, which would lead to starvation, i.e., the costly metabolism of lipids and proteins, and confer growth deficits. Arabidopsis mutants that fail to produce normal semi-crystalline starch remobilize their glucan reserves too rapidly at night and thus enter starvation (*isoamylase1* mutants; Delatte et al. 2005; Feike et al. 2016). Interestingly, a similar starvation response has been reported for plants deficient in another protein that has been hypothesized to help the crystallization of starch (ESV1; Feike et al. 2016). Although the mechanisms that regulate starch metabolism are largely unknown, it thus appears that the insolubility of starch was the key to its controlled remobilization in a diurnal cycle.

To fully understand the regulation of starch metabolism in its physiological context clearly is a Herculean task. Yet, we believe that the interplay between the insoluble nature of starch and the associated enzymatic activities is fundamental to this understanding. A theoretical approach can complement the extensive experimental work already performed, helping to rationalize starch biosynthesis in planta and produce models that can investigate quantities so far inaccessible experimentally. This refers, for example, to the specificities of enzymes acting on polymeric starch, which can only be indirectly determined and often lead to misconceptions. In this review, we first summarize essential structural features of the starch granule and then combine theoretical and experimental approaches to shed new light onto the enzymatic processes that underlie starch formation. Because of its higher biosynthetic and structural complexity, we focus on amylopectin, the major and essential component of starch.

## Starch structure and use as an energy storage

Starch consists of the two components amylopectin and amylose. Both are composed of α-1,4 linked linear glucose chains that are connected via α-1,6 linkages at branch points, but their structures differ markedly. Amylose molecules are long glucan chains with a degree of polymerization (DP) of ca. 10^2^–10^3^ that carry only a few and, in some starches, very short branches (Manners 1989; Pérez and Bertoft 2010). In contrast, amylopectin molecules consist of numerous branches that are connected in a tree-like pattern, giving rise to large molecules with a DP of ca. 10^5^ (Manners 1989; Fig. 1). Amylopectin furthermore makes up the bulk material of starch, while amylose constitutes only around 10–30% of the dry weight of wild-type starches and is dispensable (Buléon et al. 1998). Amylose is believed to fill spaces within the amylopectin matrix, increasing the density of starch and thus the efficiency by which plants can store carbohydrates (Zeeman et al. 2010). Despite being a minor component, amylose also has an impact on other properties of starch, in particular its freeze–thaw stability and digestibility in the gut, and both amylose-free and amylose-enriched starches are of high industrial value (Santelia and Zeeman 2011; Regina et al. 2015).

**Fig. 2:**
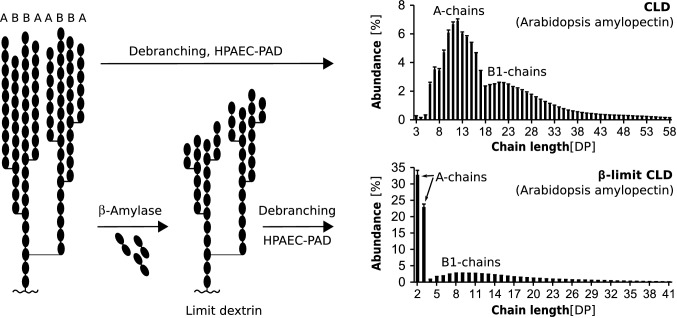
Chain-length distributions and limit chain-length distribution as a means to analyze amylopectin structure and enzyme function. Upper panel: CLDs are obtained by first enzymatically debranching the glucan to yield linear chains, separating the chains according to their length using HPAEC-PAD or another system, and then quantifying the relative peak area corresponding to each chain length. As exemplified here on amylopectin from Arabidopsis, the most abundant chain group, which peaks at DP 12, are A-chains, followed by the group of B1-chains, which peaks at DP 21. The branched substrate (left) illustrates a glucan part that has no C-chain, with letters indicating the chain category. Lower panel: To obtain a CLD of a limit dextrin, the branched glucan is treated with β-amylase (and/or starch phosphorylase) prior to debranching. β-Amylase releases maltoses (DP 2) from the non-reducing ends of the chains until approaching a branch point, i.e., it shortens all A-chains to a stub of either DP 2 or DP 3 (depending on whether the chain’s DP was even or odd). B-chains remain as fragments spanning their own attachment site to the outermost branch they carry plus one or two glucose units, again depending on the DP of the unbranched external segment. Debranching and further analysis as for CLDs provide indication about the branching pattern of the glucan, including the ratio between A-chains (represented by DP 2 and DP 3) and B-chains (primarily represented by DP > 3) and the lengths of internal (non-digested) segments of B-chains (Thompson 2000). While both CLDs and limit-CLDs are useful to compare glucans from plant mutants, and before and after their in vitro modification by enzymes, absolute values from HPAEC-PAD suffer from a slight detection bias in favor of shorter chains. This can be overcome by chain labeling and other methods (O’Shea et al. 1998; Wu et al. 2014)

The tree-like architecture is one of the most remarkable and intriguing features of amylopectin (Fig. 1). Although the fine structure of amylopectin has not been unambiguously resolved, it is widely accepted that short amylopectin chains are oriented within clusters that are connected by longer chains (French 1972). The frequency distributions of chain lengths (termed chain-length distributions or CLDs; Fig. 2) after the enzymatic debranching of amylopectins from various botanical sources are all polymodal (Hizukuri 1986). The shortest chains (peak at DP ~ 13) probably represent the so-called A-chains, which do not carry any branches themselves, followed by the different types of B-chains, which all carry at least one branch: B1-chains (peak at DP ~ 22), B2-chains (peak at DP ~ 42), B3-chains (peak at DP ~ 69), etc. (Hizukuri 1986). A- and B1-chains are believed to make up the clusters, while the longer B2- and B3-chains extend into two or three clusters, respectively. As the branch points within a cluster are furthermore enriched towards the cluster’s starting point (i.e., towards the reducing end of an amylopectin molecule), emerging linear chain segments can become sufficiently long to pair in the form of double helices. If adequately spaced, neighboring double helices can align in parallel, forming crystalline lamellae either of the tightly packed A-type (e.g., in cereal starches) or more hydrated B-type allomorph (e.g., in potato tuber starch and Arabidopsis leaf starch). The alternating arrangement of these crystalline lamellae with amorphous ones, which contain the branch points, is believed to underlie the 9–10 nm repeat, which is fundamental to all wild-type plant starches and can be observed by X-ray scattering (Jenkins et al. 1993) and electron microscopy (Kassenbeck 1978).

An alternative to the cluster model is the building block backbone model by Bertoft, in which the long chains spanning several lamellae are oriented perpendicular to the shorter chains constituting the crystalline lamellae (Bertoft 2017). Instead of forming clusters, these shorter chains would be organized as smaller building blocks that are distributed along the backbone chains. Even though this model is in line with most of the current structural data of amylopectin, the distribution of building blocks does not explain the polymodality of longer chain lengths (described above) observed in all amylopectins so far. Clearly, either model has strong implications for the underlying biosynthetic process, in particular regarding the direction of molecule growth and how a new lamella is initiated, and a mathematical model of starch biosynthesis may help to distinguish between them.

There is evidence for further levels of organization on a larger scale (Fig. 1). These levels include ‘blocklets’ (Gallant et al. 1997) and concentric ‘growth rings’ (Yamaguchi et al. 1979; Pilling and Smith 2003), in which semi-crystalline layers containing many 9–10 nm repeats alternate with amorphous zones. However, the precise nature of these structures and the mechanisms of their arrangement are not yet understood (Pérez and Bertoft 2010), so they are currently compatible with either model.

The insoluble nature of starch allows the plant to store energy in an osmotically inert and compact form. The storage efficiency of starch in plants can be compared with that of glycogen, which is the storage carbohydrate of animals, fungi, and many bacteria (Wilson et al. 2010). Glycogen is composed of the same monomers and linkages as amylopectin, but with approximately doubled branch frequency and a branching pattern which probably precludes the formation of higher-order structures (Manners 1991). As a consequence, glycogen takes the form of small water soluble particles.

The typical size of β-type glycogen particles in muscles ranges between 20 and 30 nm in diameter with a molecular mass of 10^6^–10^7^ g/mol (Gilbert and Sullivan 2014). Assuming a spherical shape, particles would range from 4.2 × 10^3^ to 1.4 × 10^4^ nm^3^, containing between 6.2 × 10^3^ and 6.2 × 10^4^ glucose monomers (based on the molecular mass of 162 g/mol monomers, which considers the loss of a water molecule during the formation of the glucosidic bond). Because of the complex and largely unknown branched structure of glycogen, it is not feasible to establish a universal correlation between the molecular size and the weight of the molecule (Gilbert and Sullivan 2014), and we therefore simply assume that the smaller particles are the lightest and the largest particles are the heaviest. Hence, for such glycogen particles, the number of glucose per volume unit would range from 1.5 to 4.4 glucose/nm^3^.

By contrast, density measurements of air-dried A- or B-type starches provide densities of 1.5 g/cm^3^ (Isleib 1957; Dengate et al. 1978), which corresponds to 9.3 × 10^−3^ mol glucose/cm^3^ or, equivalently, 5.6 glucose/nm^3^. However, cryo X-ray ptychographic tomography of fully hydrated amylose-free B-type Arabidopsis leaf starch measured a density of 1.36 g/cm^3^ (Pfister et al. 2016). Taking the assumed water content of B-type starch of ca. 27% (Pérez and Bertoft 2010) into account, this results in a considerably lower glucose density of 3.7 glucose/nm^3^, which may reflect the differences in allomorph, amylose content, or in the three-dimensional structure of dried vs. hydrated starch. Nonetheless, both values are in good agreement with the proposed measures of the crystalline unit cells. A-type allomorphs have a parallelepiped unit cell with axes of length *a* = 2.124 nm and *b* = 1.172 nm, which contains two DP 12 double helices (i.e., 24 glucose monomers) with a typical length *c* = 4.2 nm (Pérez and Bertoft 2010). Hence, in such unit cell the average number of glucose per volume unit is ca. 5.5 glucose/nm^3^, while the corresponding value for the B-type unit cells (Pérez and Bertoft 2010) is ca. 3.9 glucose/nm^3^. These values, however, are only based on the crystalline zones, not considering the amorphous ones, the presence of amylose, water, and small amounts of proteins, and lipids and phosphate groups present in starch (Pérez and Bertoft 2010). Despite these uncertainties in the measurements, they support the idea that glucose is stored with a higher density in starch than in glycogen.

In addition to starch, plants also store their assimilated carbon in other forms, serving different needs in terms of storage capacity, digestibility, and energy density. For instance, energy is stored in the form of soluble sugars such as sucrose, which can be readily metabolized within the plant, but has limitations on storage imposed by its solubility and osmotic effects. If high levels are to be stored, this requires additional means to maintain osmotic balance, such as compartmentalization of sugars between the vacuole and the apoplast in the stems of sugar cane (Welbaum and Meinzer 1990). Oils constitute another alternative, which is particularly common in seeds. Oils contain more than twice the energy per weight compared with starch; however, they require considerably more energy for their synthesis, but not all of which is regenerated when they are catabolized. Furthermore, assimilated carbon is lost as CO_2_ both during fatty acid production and when acetyl-CoA released by fatty acid β-oxidation is converted to sugars via the glyoxylate cycle. The benefits to the plant appear to outweigh these costs in some situations, for example, in terms of reducing seed weight to aid dispersal. In contrast, the metabolism of starch requires relatively little energy and does not result in the loss of assimilated carbon, which may explain its abundance and conservation within the plant kingdom. Interestingly, a few plant species also produce glycogen on occasion: in the *Azteca*-*Cecropia* mutualism, the *Cecropia* plant accumulates glycogen in Müllerian bodies, which provide a food source for hosted ants that probably are unable to metabolize starch (Rickson 1971; Bischof et al. 2013).

## How does the amylopectin structure come about? What is needed to simulate it in a model?

**Fig. 3:**
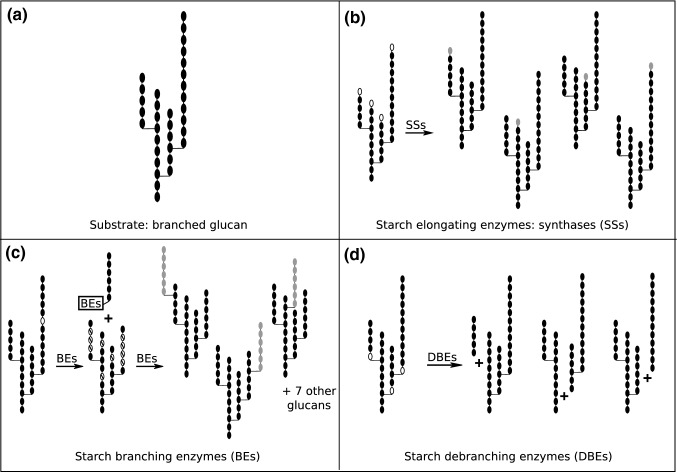
Possible glucans resulting from a single reaction of elongation, branching, or debranching on a branched substrate. **a** Small branched glucan that is used as substrate. **b** During a starch synthase (SS) reaction the glucose moiety (highlighted in gray) of an activated glucose can be added to either of the four glucoses at the non-reducing ends (in white), resulting in four possible glucans. **c** Possible branching enzyme reaction on the same small branched glucan, assuming constraints for the transferred chain segments (substrate chain DP ≥ 12, transferred segment DP = 6) and branch point placement (at least one spacing glucose between branches, reducing- and non-reducing ends not allowed). While only one α-1,4 linkage (left, in white) can serve as cut site for chain transfer, ten glucose units can serve as potential anchors for branch attachment (middle, in barred white). Three of the ten possible glucan products are shown, with transferred segments highlighted in gray. **d** A debranching reaction, hydrolyzing one of the three α-1,6 linkages (in white), can lead to three distinct glucan products. In a subsequent reaction on one of the resulting products, fewer α-1,6 linkages will be present, reducing the number of possible outcomes in every generation. We emphasize that the reactions shown are illustrative; the starch biosynthetic enzymes likely have additional specificities that define the range of substrate chains on which they act

Amylopectin synthesis involves three distinct classes of enzymatic reaction: the elongation of glucan chains by starch synthases (SSs), the introduction of branches by branching enzymes (BEs), and the selective removal of branches by debranching enzymes (DBEs; Fig. 3). It is important to conceive these as integrated activities, occurring simultaneously rather than in a sequential manner, i.e., SSs, BEs, and DBEs are present at the same time, acting on different parts of the same glucan polymer. Moreover, these reactions presumably take place at the granule surface due to restricted access to the inner parts of the polymeric glucan material, which has impact on the number of possible reaction sites and the likelihood that a particular reaction will take place. This also means that the number and shape of starch granules could play an important role as they influence the surface to volume ratio. The effect of available surface area of the substrate on the kinetics of surface-active enzymes can, under certain conditions, be modeled using Langmuir kinetics (Kartal and Ebenhöh 2013; Supplementary material; Fig. S1).

Starch synthesis additionally requires the provision of adequate glucan primers, the nature and origin of which are currently unknown. The process of granule initiation, during which soluble primers are synthesized and elaborated, differs to that of the subsequent amylopectin synthesis on the surface of an existing granule. Starch granule initiation is believed to be facilitated by a specialized protein set located in distinct sub-plastidial regions. The reader interested in these processes is re-directed to recent reviews (Seung and Smith 2019; Malinova et al. 2018).

### Chain elongation and the SS family

SSs catalyze the transfer of the glucosyl moiety from ADP-glucose to the non-reducing end of glucan chain, creating an α-1,4 glucosidic bond (Fig. 3b). The nature of this reaction may seem simple, yet even with a small branched precursor, the number of possible glucan structures after a few elongation steps is already surprisingly high. A branched glucan with *N* chains that goes through *m* elongation steps, each consisting of the random selection and elongation of one branch of the *N* branches, can result in $$\left( {\begin{array}{*{20}c} {m + N - 1} \\ {N - 1} \\ \end{array} } \right) = \frac{{\left( {m + N - 1} \right)!}}{{m!\left( {N - 1} \right)!}}$$ different molecules. While a single elongation reaction on a small branched glucan with four chains can give rise to up to four distinct products, if all chains are distinguishable (Fig. 3b), 20 randomly selected elongation steps on the same substrate can already result in as many as 1771 different glucan structures.

In addition to estimating the number of possible outcomes after the random elongation of a branched glucan, we can also predict the probability for each of these outcomes. For this, we consider *N* initial chains of the same degree of polymerisation, denoted as DP_*n*_. At each elongation step or generation, a chain among all is randomly selected and elongated. Hence, the probability for a chain of any DP to be elongated at any step is 1/*N*, and the probability for it not to be elongated is 1 − 1/*N*. After *m* elongation steps, the probability for a chain to have undergone *k* elongations, leading to a chain length of DP_*n*+*k*_, therefore is a binomial distribution:

$$P\left( {{\text{DP}}_{n + k} } \right) = \left( {\begin{array}{*{20}c} m \\ k \\ \end{array} } \right) \cdot \left( {\frac{1}{N}} \right)^{k} \cdot \left( {1 - \frac{1}{N}} \right)^{m - k}$$

**Fig. 4:**
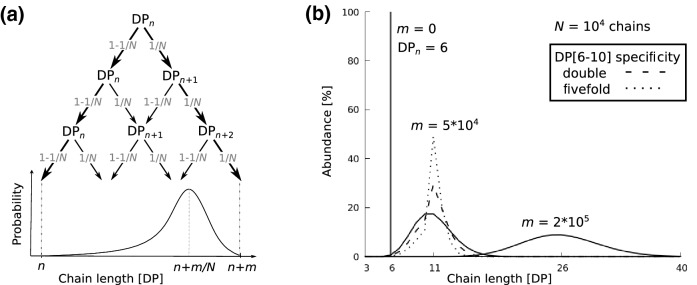
Dynamics of the chain length of a pool of *N* glucan chains through *m* recursive elongation steps. **a** Evolution of a single chain, with an initial degree of polymerization (DP) of *n*. At each elongation step or generation, the chain is randomly either elongated (with a probability of 1/*N*) or not elongated (with a probability of 1 − 1/*N*). These typical recursive attempts are known from statistics as Bernoulli trials and can be depicted by a decision tree (upper panel). The chain-length distribution (lower panel), known from statistics as mass function, illustrates the possible chain lengths achieved by the initial chain after *m* generations. The most probable chain length is *n *+ *m*/*N*. The extreme and most unlikely cases are elongation at each generation and no elongation at each generation, which occur with the probabilities of (1/*N*)^*m*^ and (1 − 1/*N*)^*m*^, respectively. **b** Stochastic simulations illustrate the random elongation of glucan chains from an initial monodisperse pool of 10^4^ chains of DP 6. Chain-length distributions are recorded after 5 × 10^4^ and 2 × 10^5^ generations. Simulations either mimic the action of an elongating enzyme without any chain-length specificity (i.e., all chains are elongated with the same probability) or with a double or fivefold higher probability to elongate short chains of DP 6–10 compared with other chain lengths. The latter simulates potential chain-length specificity of a starch synthase. While after 5 × 10^4^ generations the distribution with chain-length specificity becomes narrower, the introduced specificity has no impact on the distribution after 2 × 10^5^ generations as the chains DP 6–10 have become depleted, i.e., the curve completely overlaps with that without specificity

The resulting probability curve for the length of an individual chain (Fig. 4a, lower panel) is asymmetric due to the bias between the probabilities for elongation and no elongation as soon as *N* > 2. This curve also equals the average chain-length distribution from an infinite number of glucan chains that have, in total, undergone *m* elongation steps. The mean degree of polymerization of glucan chains after *m* generations, noted $\overline{\text{DP}} \left( m \right)$, is equal to the initial degree of polymerisation (*n*) plus the number of elongation steps (*m*) divided by the total number of chains (*N*):

$$\overline{\text{DP}} \left( m \right) = n + \frac{m}{N}$$

When recording the evolution of an initial monodisperse pool of 10^4^ linear glucan chains of DP 6 after 5 × 10^4^ and 2 × 10^5^ elongation steps or generations (Fig. 4b), the average chain length increases with the number of elongations: it reaches DP 11 after 5 × 10^4^ generations, corresponding to 5 elongations per chain on average, and DP 26 after 2 × 10^5^ generations, corresponding to 20 elongations per chain on average. One should also remark that the system we investigate is not under stationary conditions, and in particular the chain-length distribution is not stationary, but changes with the number of generations. As chains are randomly selected for every elongation, subsequent elongation steps are independent from each other. This fundamental notion from statistics is conceptually close to what is referred to as distributive or non-processive elongation, i.e., the elongating enzyme releases its target chain after every elongation. As a consequence of the random selection, the initial peak of monodisperse chains turns into a polydisperse distribution which becomes wider with an increasing number of elongation steps. In this example, the likelihood for elongation is equal for every chain, i.e., the elongating SS would not display any chain preferences, neither in terms of chain’s length, its branching level, and position within the glucan or its potential engagement in secondary or higher-order structures. However, in reality, SSs do have preferences for the elongation of certain chains, but these preferences are not trivial to determine experimentally and there are multiple SS isoforms that need to be considered.

Amylopectin biosynthesis involves four SS isoforms: SSI, SSII, SSIII, and SSIV. Plant species and tissues vary in the apparent contributions of the individual SSs, which undoubtedly underlies some of the differences observed in amylopectin structure (Fujita 2014). Further, there are additional SSs that may impact amylopectin synthesis: the partly processive granule-bound starch synthase (Edwards et al. 1999; Cuesta-Seijo et al. 2016), which is primarily required for amylose production, and at least two putative SSs—SSV and SSVI—the functions of which are as yet unknown (Liu et al. 2015; Van Harsselaar et al. 2017).

SSI to SSIV belong to the glucosyl transferase family 5 (CAZy database; Lombard et al. 2014) and share a highly conserved catalytic domain in their C-terminal region. Every class of SS, however, differs in the domain structure of appended N-terminal amino acid stretches. While SSI has a short N-terminal extension without any predicted motifs, SSIII has a long N-terminal extension containing three carbohydrate-binding modules, interspersed with coiled-coil motifs (Valdez et al. 2008; Wayllace et al. 2010). This N-terminal variability probably helps confer the functional distinction of SSs, either by intrinsically altering their specificity or by binding to interaction partners. Put simply, SSI appears to be particularly important for the elongation of the shortest amylopectin chains that directly derive from BE action, SSII elongates them further to reach their final length within a cluster, and SSIII seems to be key to the synthesis of long cluster-spanning chains (Pfister and Zeeman 2016). The unique function of SSIV lies in the initiation of starch granules rather than amylopectin synthesis, although it is capable of synthesizing small amounts of insoluble glucans in the absence of the SSI, SSII, and SSIII (Roldán et al. 2007; Szydlowski et al. 2009).

The functional classification of SSs typically derive from the chain-length analysis of starches from mutant plants lacking single isoforms. These analyses, however, will only reveal protein functions that are not taken over by other isoforms, potentially failing to depict the actual major activity of SS if functional redundancies exist. Alterations in starch structure may also arise from indirect effects, such as the deregulation of other genes in a given mutant background (as observed for *ssIII* mutants; Cao et al. 1999; Fujita et al. 2007; Ral et al. 2006). In the event of complex formation between starch biosynthetic proteins, mutant phenotypes may also reflect the altered activities of previously interacting proteins when present in their monomeric forms (Tetlow and Emes 2011; Pfister and Zeeman 2016).

Functional interdependencies may be exposed using plant lines deficient in multiple isoforms. Indeed, the analysis of several mutant combinations among SSI, SSII, and SSIII in Arabidopsis revealed that SSII and SSIII are partly redundant for the elongation of intermediate length chains (Zhang et al. 2008), but SSI, SSII, and SSIII are not dependent on each other for chain elongation (Szydlowski et al. 2011; Pfister et al. 2014). The CLDs of glucans made by the reduced enzymatic set remaining in multiple SS mutants tend to resemble that of glucans made from the remaining SS(s) in conjunction with a BE, synthesized either in vitro (Brust et al. 2014) or using a heterologous system as a platform for recombinant protein expression and glucan synthesis (Pfister et al. 2016). For instance, glucans made from BEs and SSI as the sole or predominant synthase activity, either in Arabidopsis (Zhang et al. 2008; Szydlowski et al. 2011; Pfister et al. 2014; Pfister and Zeeman, unpublished observations) or in vitro (Brust et al. 2014), are enriched in short chains of DP 7–9 and depleted in longer chains, supporting the idea that SSI preferentially synthesizes short glucan chains of ca. DP 8.

In vitro approaches also offer the prospect of providing direct estimates of kinetic constants and specificities, including *K*_M_, *k*_cat_, and chain-length preferences, from monitoring the reactivity of SSs towards defined substrates such as malto-oligosaccharides. The recent in vitro analysis of recombinant barley SSs provided a valuable source of such substrate recognition and catalysis information (Cuesta-Seijo et al. 2016). For SSIV, thought to be involved in granule initiation by acting on low molecular weight glucans, such kinetic information can be compared with its physiological action. However, when an enzyme’s physiological action is on polymeric starch, kinetic information for substrates like malto-oligosaccharides, glycogen, or even solubilised amylopectin might not give a full picture. It is unlikely that soluble glucans will fully reflect the partly insoluble nature of the surface of a starch granule where tightly packed, branched polymers likely result in molecular crowding and high local viscosities. Thus, the extent to which in vitro characteristics of SSs hold true when they act in vivo is unclear. That said, precise assays with realistic starch-like substrate surfaces are not trivial to design or execute, highlighting the need for modeling approaches that complement experiments to achieve physiologically realistic simulations of starch granule biogenesis (O’Neill et al. 2014; O’Neill and Field 2015).

Translating features like the experimentally observed preferential synthesis of chains around DP 8 by SSI into an elongation preference for DP 6–10 in numerical simulations can have a marked impact on the resultant CLDs (Fig. 4b). Importantly, when chain-length specificity is introduced, the likelihood for a chain to be selected for elongation depends on the chain length, but the chain selection itself remains random. As shown in our first example above, without specificity, the distribution after 5 × 10^4^ elongation steps is centered on DP 11. When introducing specificity for chains DP 6–10, the distribution becomes narrower, which can be explained as follows: with increasing probability for the elongation of DP 6–10, these chains become more likely to be elongated, eventually leading to their depletion. At the same time, chains of DP 11, where the likelihood for elongation drops, become more abundant. However, when further increasing the number of generations, chains of DP < 11 become even more depleted such that fewer and fewer chains remain to which the specificity applies, continuously diminishing the effect of the specificity. This is illustrated in the observation that after 2 × 10^5^ elongation steps, the CLDs with specificity is indistinguishable to that without (Fig. 4b).

This example demonstrates how easily CLDs can be misinterpreted. Clearly, a high number of elongation steps can mask chain-length specificities if the chains affected by the specificities become depleted. At the same time, however, peak shifts in CLDs do not necessarily stem from chain-length specificities, but can simply result from changes in reaction times when stationary conditions are not reached, as exemplified in the curve differences after 5 × 10^4^ and 2 × 10^5^ elongation steps (Fig. 4b). Furthermore, the simulations indicate that the specificity of SSI is more complex than initially assumed, since the elongation preference for DP 6–10 did not yield a CLD centered on DP 8. These considerations show how statistics and modeling can complement experimental approaches to provide quantitative interpretations of experimental observations, thus providing insights into quantities like chain-length specificities, which can hardly be determined with either approach alone.

### Introduction of branches by BEs—increasing the complexity

Branching enzymes introduce branches by a cut-and-paste trans-glucosylation mechanism: they first cleave an α-1,4 glucosidic linkage and then ligate the reducing end of the cut chain either to the same or another chain (Viksø-Nielsen et al. 1998; Rydberg et al. 2001) via an α-1,6 linkage (Fig. 3c). Interestingly, this reaction is energetically favored as the Gibbs energy associated with the conversion of maltose (α-1,4 linked glucoses) to isomaltose (α-1,6 linked glucoses) is negative: measuring the Gibbs energy released by glucan hydrolysis (Tewari and Goldberg 1989)⁠ results in ΔG^0^ ≈ − 8.44 kJ/mol, and computing this quantity using the component contributions method (Noor et al. 2013) yields ΔG^0^ ≈ − 6.0 ± 3.5 kJ/mol.

The BE reaction can potentially result in an enormous number of possible outcomes: while SSs can attach the new glucose unit only to the non-reducing end of a chain (i.e., one possible position per chain), BEs theoretically have many possibilities regarding the cleavage site of the donor chain and the site onto which the chain is attached. Data from in vitro studies suggest relatively loose constraints, such as ca. DP 12 as the minimal length of a chain to become donor for BE action and DP 6 as the shortest transferred chain segment (Fig. 3c; Nakamura et al. 2010; Sawada et al. 2013, 2014). Operating alone on a glucan substrate, BEs would deplete their own glucan substrate by removing α-1,4 bonds and shortening the chains, continuously reducing the number of subsequent possible outcomes with each reaction. In reality, however, BEs work in conjunction with SSs, mutually producing each other’s glucan substrates: SSs create α-1,4 bonds for BEs to act on and BEs synthesize non-reducing ends for elongation by SSs.

Assuming only very basic constraints for both enzymatic steps of a single chain transfer by a BE, we can estimate the total number of possibly resulting glucan structures as follows. Here we assume that all branches are distinguishable, which tends to be true for complex biological polysaccharides like amylopectin. We denote by *Ω*_C_ the number of positions where the BE can cleave an α-1,4 bond, assuming that (1) only chain segments with exactly DP 6 (counted from the non-reducing end) can be cut, (2) these segments must not carry any branches themselves, and (3) the remaining chain that is left behind is of DP ≥ 6. With these constraints, a chain can be cleaved only at one position at most, and *Ω*_C_ simply equals the number of glucan chains with DP ≥ 12 whose outermost branch is at least 7 glucose units distant from the non-reducing end.

In addition, we denote by *Ω*_P_ the number of possible paste positions within the glucan, for which we—as a strong simplification—only exclude the glucoses whose C6 is already engaged in forming a branch point. Finally, we denote by *Ω* the total number of resulting glucans for a single chain transfer. It corresponds to any combination for the hydrolysis of an α-1,4 bond, followed by the creation of a new α-1,6 branch point, and can be estimated as:

$$\varOmega = \varOmega_{\text{C}} *\varOmega_{\text{P}}$$

These minimal constraints lead to 24 possible glucans resulting from a single BE reaction on the example glucan from Fig. 3, thus strongly exceeding that of the SS reaction (note that in Fig. 3c additional constraints for branch point placement are applied). The value for *Ω* indeed has the potential to become even larger as the number of chains increases. The plethora of theoretical branching possibilities, however, needs to be corrected by the fact that BEs have additional constraints, as considered below. In reality, the distribution of branch points in amylopectin appears to be tightly governed and not homogenous; rather regions with relatively high branch frequency alternate with relatively unbranched regions, underlying the semi-crystalline nature of starch (Thompson 2000). The exact contribution of BEs in the ultimate positioning of branches in starch granules is difficult to decipher, since it is also influenced by branch removal by debranching enzymes and the elongation of chains by SSs. Nevertheless, the placement of branches by BEs likely sets the basis for the final amylopectin structure, necessitating the incorporation of their in vivo action in a model.

Starch BEs occur in plants as only two isoforms, BEI and BEII, which belong to glycoside hydrolase family 13 (superfamily of α-amylases) and share their domain structure over the whole protein (reviewed in Tetlow and Emes 2014). This simplicity, however, is overshadowed by the challenge of assessing their actual activity at a resolution sufficient to deduce their specificities. Current structural analyses of glucans allow determining the chain lengths and branch numbers, but not their exact positions within a glucan.

A useful means to monitor BE activity is to compare CLDs (Fig. 2) of moderately branched glucans before and after their in vitro modification by a BE. Here, chain lengths that decrease in absolute abundance inevitably served as donor for the BE, while those that increase in abundance either constitute transferred segments or, less frequently, represent the remainder of chains after a part has been transferred (Nakamura et al. 2011). This technique has allowed the minimal lengths for donor and transferred chains of BEs to be defined as ca. DP 12 and DP 6, respectively (Nakamura et al. 2010; Sawada et al. 2013, 2014). However, where the new α-1,6 linkages are formed is not revealed because the debranching performed prior to CLD analysis leaves no trace.

Some information on branching patterns can be obtained from limit-CLDs, where all external linear chain segments are enzymatically degraded by an exo-acting enzyme, typically starch phosphorylase and/or β-amylase, prior to debranching. Degradation stops as the enzymes approach the branch points, leading to a glucan skeleton in which all A-chains remain as short stubs and the B-chains are shortened until close to the outermost branch they carry (Fig. 2). Subsequent CLD analysis then reveals the number of A-chain stubs and the lengths of the residual B-chain segments. This allows measurement of the ratio between A- and B-chains, the average number of branches that B-chains carry, and the average external (degradable) chain lengths, among others (Fig. 2; Thompson 2000). Although useful, this technique still fails to depict the distances between some branch points. Since it only measures the distance between the outermost branch a B-chain carries and its own attachment site, branches can become hidden in between others. Nevertheless, very short B-chains in limit-CLD analysis most likely carried only a single branch that was close to the B-chain’s own attachment, indicating that the two branch points were in close proximity.

When applied to glucans before and after modification by rice BEs, limit-CLD analysis indicated that branches can be placed at several positions within a chain, with no obvious preference for placing them onto A- or B-chains (Sawada et al. 2014). This is consistent with limit-CLDs of various plant amylopectins, which show a relative broad range of B-chain remnants (Bertoft et al. 2008; Fig. 2). However, amylopectin typically does contain low amounts of very short B-chain remnants, suggesting that the branches can be placed as close as two glucose units away from the previous branch point (i.e., with one free glucose unit in between; Bertoft et al. 2008; Fig. 3c).

Taking this putative minimal distance between branch points into account, we can re-estimate the total number of resulting glucans for a single chain transfer. As the number of outcomes from the hydrolysis of the α-1,4 linkage (Ω_C_) is not affected, we only reconsider the possibilities for the position of the new branch point. In addition to the glucoses engaged in α-1,6 bonds, we now also exclude one spacing glucose above and below each branch point, all non-reducing ends and the reducing one. When performed on the example glucan from Fig. 3c, these additional constraints drastically reduce the number of possible paste positions *Ω*_P_ from 24 to 10.

This number most likely still overestimates the actual number of possibilities due to the potential additional constraints that have not been captured experimentally. The transferase action of a BE may happen locally within the glucan molecule, as BEs probably bind the donor chain (from which the transferred segment derives) and the acceptor chain simultaneously—this would help prevent BEs from transferring the cut chain segment to water (i.e., simply hydrolyzing the glucan). Indeed, crystal structures of rice and cyanobacterial BEs suggest that glucans are not only bound to the active site, but also to additional starch-binding sites at the protein surface, although the actual intermediate with at least one covalently bound chain has not been captured (Chaen et al. 2012; Hayashi et al. 2017). Furthermore, the accessibility of potential cleavage and paste sites of BEs could be reduced by the formation of double helices and crystalline lamellae, i.e., both *Ω*_C_ and *Ω*_p_ would drop.

Again, modeling offers the possibility to implement these aspects. Monitoring the proximity between chains during the simulation allows to assess whether simultaneous binding of donor and acceptor chains could occur, which could then be translated into probabilities for the selection of attachment sites by a BE. These probabilities could also consider the likelihood of chains to be engaged in a double-helical structure by implementing experimentally derived chain-length requirements for the formation of such structures (see below; Gidley and Bulpin 1987; Pfannemüller 1987). Given the potential importance of these considerations—not just for BEs, but for all the starch biosynthetic enzymes—implementation of higher-order structure formation into a model for starch biosynthesis would represent an important theoretical and conceptual advance.

### Branch removal by debranching enzymes—a balance between tailoring and degradation

The possible reaction sites for DBEs are easily defined as the α-1,6 linkages and, as DBEs hydrolyze these linkages, they deplete their own substrate (Fig. 3d). If the hydrolysis of branch points within a glucan was unrestricted, the outcome would eventually always be a pool of linear malto-oligosaccharides. Clearly, this is not what happens during in vivo amylopectin biosynthesis, where debranching activity is widely believed to facilitate the efficient crystallization of the nascent glucan, rather than completely degrading it (Ball et al. 1996). This implies that debranching activity is tightly governed, but how this is accomplished remains enigmatic.

Plant genomes encode four DBE genes—three isoamylases (ISA1, ISA2, and ISA3) and one limit-dextrinase (also called pullulanase). ISA1 and ISA2 have been implicated in starch biosynthesis under normal conditions and are the focus here. ISA3 and limit-dextrinase are primarily engaged in the process of starch breakdown (Wattebled et al. 2005; Delatte et al. 2006), though they can modulate starch biosynthesis, in particular when ISA1 and/or ISA2 are missing (Wattebled et al. 2008; Streb et al. 2008; Fujita et al. 2009). Like BEs, ISA1 and ISA2 belong to glycoside hydrolase family 13; they share the overall domain structure of α-amylases including the carbohydrate-binding module (Pfister and Zeeman 2016). ISA2 carries point mutations in several key amino acids within its active site, which render it catalytically inactive (Hussain et al. 2003). ISA1 and ISA2 together form an active heteromultimeric complex, in which ISA1 serves as the catalytic subunit, while ISA2 may help stabilization and direct the specificity of the complex (Hussain et al. 2003; Delatte et al. 2005; Wattebled et al. 2005; Sundberg et al. 2013; Facon et al. 2013). In addition to the heteromultimeric form, cereals and Chlamydomonas also display a homomultimeric ISA1 activity (Utsumi and Nakamura 2006; Kubo et al. 2010; Sim et al. 2014). Hereafter, we refer to both activities as “ISA”, since the functional distinction between the two is not well defined.

Plant mutants deficient in ISA typically accumulate water soluble glucans in addition to, or instead of, starch (Pan and Nelson 1984; Nakamura et al. 1996; Mouille et al. 1996; Zeeman et al. 1998). These soluble glucans have a structure similar to that of glycogen and have thus been termed phytoglycogen. The presence of phytoglycogen in *isa* mutants has underpinned the view that ISA ‘trims’ and thereby facilitates the crystallization of glucans (Ball et al. 1996). According to this model, ISA selectively hydrolyses excess or wrongly positioned branches that would otherwise lead to steric hindrances and prevent the formation of a new crystalline lamella. Although widely accepted, the molecular basis of the selective action of ISA inferred by this model is not understood. The release of chains by ISA cannot be followed in planta, as such chains do not accumulate—presumably they are rapidly processed by other glucan-metabolizing enzymes such as β-amylase (see below). Also, in vitro debranching assays on branched substrates have not demonstrated clear chain specificities for ISA, except that the very short chains that normally occur during starch degradation are poor substrates (Delatte et al. 2006; Sim et al. 2014; Kobayashi et al. 2016).

In principle, excessive debranching could be prevented by maintaining a tight balance between BE and ISA activities. However, this seems unlikely. Mutant or transgenic plants with strongly reduced amounts of BE still produce substantial amounts of glucans that are insoluble and branched, albeit with markedly fewer branches (Klucinec and Thompson 2002; Regina et al. 2010), presumably as a primary effect from lowered BE activity. In contrast, ISA was found to act in a degradative fashion on highly branched substrates. ISA expression in the glycogen-producing *E. coli* and *S. cerevisiae* cells did not lead to the formation of insoluble, starch-like glucans, but drastically decreased their glucan content (Sundberg et al. 2013; Pfister et al. 2016). Likewise, the Arabidopsis *ssII ssIII* double mutant produces only tiny amounts of glucans enriched in short chains, but the amounts are considerably higher when ISA is absent (Pfister et al. 2014). It hence appears in all three systems that ISA debranches short-chain-containing glucans into linear chains that are ultimately turned over.

Based on these data, it is plausible that ISA specificity relevant for its role in amylopectin trimming is linked to the structure of the glucan substrate made by the SSs and BEs. One possibility is that branch points between chains that form secondary structures (i.e., a double helix) may be inaccessible to ISA, whereas branches where chains are unpaired might be accessible. Accordingly, the association of glucan chains (and their subsequent crystallization) would prevent excessive branch point hydrolysis. If trimming by ISA helps linear chain segments to associate, its action would be self-limiting and would prevent further debranching. This concept requires that the glucan is generally competent to crystallize, i.e., SSs and BEs have endowed it with linear chain segments sufficiently long to form double helices (most chains DP ≥ 10) and more-or-less adequately spaced branches to allow double helices to associate into crystalline lamellae (Gidley and Bulpin 1987; Pfannemüller 1987; O’Sullivan and Perez 1999). When faced with glycogen-like glucans that do not meet these criteria, even upon debranching, ISA may simply continue to hydrolyze branches. A specific reactivity towards non-structured areas would be consistent with the crystallographic structure of Chlamydomonas ISA1, which suggested that its substrate-binding groove can accommodate a single chain, but not a double helix (Sim et al. 2014). These considerations again highlight the need to incorporate the formation of secondary and tertiary structures into a model to realistically simulate the biosynthetic processes.

The question remains as to what extent debranching occurs under normal conditions—how many of the branches introduced by BEs require removal for efficient amylopectin crystallization? The low abundance of malto-oligosaccharides probably does not reflect the number of released chains as they are likely turned over at a high rate. The concerted action of amylases and disproportionating enzyme (D-enzyme) may rapidly convert malto-oligosaccharides into glucose and maltose molecules that could be exported and further metabolized in the cytosol, as observed during nocturnal starch degradation (Critchley et al. 2001; Niittylä et al. 2004). At the same time, malto-oligosaccharides released by ISA may also be utilized by disproportionating enzyme and starch phosphorylase, which have been found to physically interact in the rice endosperm and to cooperatively synthesize longer malto-oligosaccharides in vitro (Hwang et al. 2016). Malto-oligosaccharides could also be elongated by SSs, since oligosaccharides with a DP ≥ 2 have been shown to serve as substrate for SSs activity in vitro (Cuesta-Seijo et al. 2016). Elongated glucan chains could then be re-incorporated by BE chain transfer or constitute a starting point for a new amylopectin or amylose molecule.

In *S. cerevisiae* strains engineered to express combinations of starch biosynthetic genes from Arabidopsis, turnover of released malto-oligosaccharides is probably lower: these yeast lines were purged of the endogenous glycogen metabolic machinery and lack the capacity to metabolize malto-oligosaccharides other than by their elongation and elaboration into branched glucans (Pfister et al. 2016). In the yeast strain expressing the whole known complement of amylopectin-biosynthetic genes, ca. 10% (in weight) of the glucans took the form of linear malto-oligosaccharides. Taking also the average chain lengths of the different glucans into account indicated that around 15% (in molar terms) of the branches had been released. However, the actual number in plants may be lower, since the yeast strain still produced soluble glucans that may have been more susceptible to debranching by ISA. Nevertheless, the observations for these engineered yeast strains represent an excellent starting point to develop an integrated and predictive theoretical model of starch biogenesis.

## Conclusions

Understanding how plants synthesize starch is a challenging task, yet essential for the rationalized and efficient modification of starch crops towards in-planta production of starch with desired functionalities for industrial end uses. Primary challenges include the plethora of enzymes involved and their inevitable interplay when building, transforming, or even depleting each other’s glucan substrate, and the technical hurdles to determine glucan fine structure at high resolution. Consequently, enzymes have often been studied alone rather than in conjunction with others and using monodisperse linear chains as substrates, which may only partly reflect the enzymes’ functions when acting on semi-crystalline starch.

Several of these difficulties can be overcome by mathematical modeling. As it offers theoretical methods, it can mimic biological phenomena based on defined and controlled assumptions, allowing vigorous hypothesis testing. A model can also address aspects that are inherently difficult to measure experimentally, such as the potential impact of starch crystallinity on the accessibility of reaction sites for enzymes. Such an impact has been known for years with respect to starch degradation, where phosphorylation of glucose residues to disrupt the tight packing of glucan chains is crucial for the efficient amylolytic attack by β-amylases (Ritte et al. 2004; Zeeman et al. 2010). In starch biosynthesis, however, understanding the effect of crystallinity and substrate availability on enzyme activity requires considering multiple factors, including the dynamics of the formation of secondary or higher-order structures concomitant with glucan synthesis. Notably, many aspects underlying glucan crystallization are hardly understood—we do not know whether they are spontaneous processes or facilitated by proteins, whether they are temporarily or spatially controlled, and how they are influenced by the local environment.

The scope of fundamental questions still pending for starch biosynthesis that can be tackled with a modeling approach is much wider than the cases illustrated here. These include the production of amylose and its incorporation into the granular matrix, the supply of ADP-glucose, and the formation of complexes between starch biosynthetic proteins, which can impact on an enzyme’s specificity, reaction radius, and binding affinities, among others. Regarding the formation of higher-order structures, many of the associated parameters are challenging to determine experimentally. Yet, these challenges highlight the potential synergy between experimentation and simulation: since the difficulties in unambiguously defining parameters experimentally necessitate validation tests, these critical evaluations present an opportunity to challenge current views and guide experimental design. In other words, conceptualizing the discovery process in terms of a synthetic biology design-build-test-learn cycle helps refine both model and experiment in an inter-dependent manner, ultimately advancing our understanding of the underlying biological process.

## Electronic supplementary material

Below is the link to the electronic supplementary material.

Supplementary material 1 (DOCX 20 kb)

Supplementary material 2 (EPS 571 kb)

## Abbreviations

ADP-glucose: Adenosine diphosphate glucose
BE: Branching enzyme
CLD: Chain-length distribution
DBE: Debranching enzyme
DP: Degree of polymerisation
HPAEC-PAD: High-performance anion exchange chromatography coupled with pulsed amperometric detection
ISA: Homomeric isoamylase 1 enzyme or heteromeric isoamylase 1-isoamylase 2 enzyme
SS: Starch synthase
