# Supplementary material for: Theoretical and experimental approaches to understand the biosynthesis of starch granules in a physiological context
Source: Photosynth Res. 2020 Jan 18;145(1):55–70. doi: 10.1007/s11120-019-00704-y (PMC7308250; doi:10.1007/s11120-019-00704-y)
Supplement: Supplementary file 1 — Supplementary material 1 (DOCX 20 kb) [file 11120_2019_704_MOESM1_ESM.docx]

**Supplementary Material**

The Langmuir adsorption model describes the adhesion of particles of an ideal gas on a surface, assuming isothermal conditions (Fig. S1, upper panel). This model, developed in the early twentieth century, is particularly suitable for the characterisation of the dynamics of surface-active enzymes (Kartal and Ebenhöh, 2013). Just like the Michaelis-Menten kinetics, the Langmuir kinetics has been further elaborated with different versions of the model, which include considerations on competitive adsorption. Here we focus on the derivation of the kinetics for a single adsorbate. The model is based on a number of assumptions whose accuracy depends very much on the system studied. The surface is assumed to be a perfectly flat and homogeneous plane, made of identical and independent sites, which can bind, at most, one particle each. The adsorbed particles are supposed to be immobile and behave independently. The adsorption of the enzyme E on the surface S is described as a reaction that forms the complex ES and is characterised by an equilibrium constant *K_eq_*, which is the ratio of the association rate constante *k_a_* over the dissociation rate constant *k_d_* (Fig. S1, upper panel). The association rate, denoted *A,* can then be expressed as the association rate constant, multiplied by the concentration of free enzyme in solution around the surface, and the fraction of unoccupied binding sites: $A=k_{a}\left[ E \right]\left( \frac{\left[ S \right]}{\left[ S \right]+\left[ ES \right]} \right)$

Similarly, the dissociation rate, denoted *D*, can be expressed as the dissociation rate constant, multiplied with the fraction of occupied sites: $D=k_{d}\left( \frac{\left[ ES \right]}{\left[ S \right]+\left[ ES \right]} \right)$.

As a complex ES is formed by the association of an enzyme on the surface, and lost by the dissociation of the enzyme, we can write the variation of the complex concentration over time as:

$$\frac{d\left[ ES \right]}{dt}=A-D\Leftrightarrow\frac{d\left[ ES \right]}{dt}=k_{a}\frac{\left[ E \right]\left[ S \right]}{\left[ S \right]+\left[ ES \right]}-k_{d}\frac{\left[ ES \right]}{\left[ S \right]+\left[ ES \right]}$$

We focus on the steady state, which is reached when the concentration of complex is invariant over time: $\frac{d\left[ ES \right]}{dt}=0$. Hence, $k_{a}\frac{\left[ E \right]\left[ S \right]}{\left[ S \right]+\left[ ES \right]}-k_{d}\frac{\left[ ES \right]}{\left[ S \right]+\left[ ES \right]}=0\Leftrightarrow\left[ S \right]=\frac{\left[ ES \right]}{K_{eq}\left[ E \right]}$ with $K_{eq}=\frac{k_{a}}{k_{d}}$.

The system is characterised by the fraction of occupied sites, knowing that the total concentration of sites, [S_tot_], is simply the sum of the concentration of the occupied, [ES], and unoccupied, [S], sites.

Therefore, $\left[ S_{Tot} \right]=\left[ S \right]+\left[ ES \right]\Leftrightarrow\left[ S_{Tot} \right]=\frac{\left[ ES \right]}{K_{eq}\left[ E \right]}+\left[ ES \right]\Leftrightarrow\frac{\left[ ES \right]}{\left[ S_{Tot} \right]}=\frac{K_{eq}\left[ E \right]}{1+K_{eq}\left[ E \right]}$. The latter equation describes the system and is called the Langmuir isotherm. When plotted (Fig. S1, upper panel) it exhibits two typical regimes: for enzyme concentrations lower than *K_eq_,* the surface coverage is limited by the number of enzymes surrounding the surface and by their ability to bind to the surface, quantified by *K_eq_*. In this regime, the surface available for binding does not play a role in the total number of sites occupied by the enzymes. Contrarily, for enzyme concentrations much greater than *K_eq_,* the surface is saturated by enzymes and both their concentrations and their ability to bind have no impact on the process. The available surface area, however, is the key parameter, and the number of enzymes bound is proportional to the surface size.

For a spherical granule, the available surface can be very simply estimated as $S=4\pi R^{2}$, with *R* the radius of the granule. And the surface of a single granule, *S_1_,* can be compared to that of several smaller and identical granules, *S_n_*, that contain the same total amount of glucan (Fig. S1, lower panel). As we equal the volume of the large granule, *V_1_,* to the total volume of the smaller granules, *nV_n_,* we can deduce the relation between the radii of the small, *R_n_*, and the large, *R_1_*, granules: $V_{1}=nV_{n}\Leftrightarrow\frac{4}{3}\pi R_{1}^{3}=n\frac{4}{3}\pi R_{n}^{3}\Leftrightarrow{R_{n}}^{2}=\left( \frac{1}{n} \right)^{\frac{2}{3}}R_{1}^{2}$.

So, the surface available for binding on *n* smaller granules can be expressed with the radius of the large granule *R_1_*: ${nS}_{n}=n4\pi R_{n}^{2}=n4\pi\left( \frac{1}{n} \right)^{\frac{2}{3}}R_{1}^{2}$, which leads to: ${nS}_{n}=n^{\frac{1}{3}}S_{1}$, and allows to conclude that the available surface increases with the cubic root of the number of identical granules that compose a given volume.

**Fig. S1: Enzyme association/dissociation dynamics at the granule surface and impact of the granule number on the surface availability. The dynamic attachment and detachment of surface-active enzymes can be described by the Langmuir kinetics (upper panel). The steady state condition is reached when the concentration of enzyme associated to the surface is invariable over time. Then, the system can be characterised by the fraction of occupied sites [ES]/[S_Tot_], which solely depends on the enzyme concentration [E], and the equilibrium constant K_eq_. For enzyme concentrations lower than K_eq_, the intrinsic attachment vs detachment property of the enzyme (K_eq_ = k_a_/k_d_) is limiting, and increasing this ratio results in higher occupation of the surface. Contrarily, for very high concentration of enzyme ([E]>>K_eq_) the surface saturates and limits the number of enzymes than can attach to it. In this regime, the number of enzymes attached to the surface is proportional to the surface area. Considering an ideal spherical granule and a fixed volume of glucan, the surface available for enzyme attachment depends on the number of granules. Several small granules offer more surface than a single, although bigger, granule (lower panel).**
